# Effects of Gibberellin Pre-Treatment on Seed Germination and Seedling Physiology Characteristics in Industrial Hemp under Drought Stress Condition

**DOI:** 10.3390/life12111907

**Published:** 2022-11-16

**Authors:** Guanghui Du, Hanxue Zhang, Yang Yang, Yinhong Zhao, Kailei Tang, Feihu Liu

**Affiliations:** Institute of Resource Plants, Yunnan University, Kunming 650500, China

**Keywords:** *Cannabis sativa* L., gibberellins, seed germination, seedling physiology, drought stress

## Abstract

The present study aimed to explore the effects of exogenous gibberellins (GAs) on seed germination and subsequent seedling growth of hemp (*Cannabis sativa* L.) under drought stress. Seeds of two industrial hemp cultivars i.e., ‘Yunma 1’, (YM) and ‘Bamahuoma’, (BM) were treated with different concentrations of GA_3_ solution (0, 200, 400, 600, 800 mg/L) at 20 °C for 8 h. The effect of pre-treatment was assessed on germination characteristics and physiological indexes on subsequent exposure to drought stress using 20% (*m/v*) polyethylene glycol (PEG) for 7 days. The results revealed that seed germination in hemp was sensitive to drought stress, as the germination indexes (germination rate and germination potential) decreased significantly, and seedling growth (hypocotyl length and radicle length) was impeded under 20% PEG-6000 condition. GA_3_ pre-treatment affected germination rate, germination potential, hypocotyl length and radicle length. With increasing GA_3_ concentration, these indexes first increased and then decreased. For seedling physiology characteristics in hemp, GA_3_-pretreatment remarkedly increased the osmotic regulating substances (soluble sugar and soluble protein contents) and the activities of antioxidant enzymes (SOD, superoxide dismutase and POD, peroxidase), while sharply decreased the lipid peroxidation (malondialdehyde, MDA) in seedlings grown under PEG-6000 induced drought stress. These results suggested that seeds pre-treated with GA_3_ could enhance the drought tolerance of hempseeds, and the optimal effect of GA_3_ for seed pre-treatment of YM and BM could be obtained when the concentration of GA_3_ solution reached 400 mg/L and 600 mg/L, respectively.

## 1. Introduction

Hemp (*Cannabis sativa* L.), belonging to family Cannabaceae, is an annual dioecious herbaceous plant in nature. The content of tetrahydrocannabinol (THC) <0.3% in fresh flowers and leaves is internationally defined as industrial hemp in China. In recent years, industrial hemp has been widely used in textile industry, paper production, medicine, food, construction industry and new energy, etc., as an economic crop with extremely high comprehensive utilization value [1,2].

Drought is one of the most common natural disasters in agricultural production, which seriously affects crop yield and quality. With the aggravation of water shortage and global warming, the growth of plants will be subjected to more severe drought stress in the future [3]. As the starting point of plant growth, seed germination is the most important stage of plant life history, which plays a decisive role in subsequent seedling establishment and plant growth. The process of seed germination is controlled by many environmental factors, such as moisture, temperature, oxygen, etc. During germination, drought stress is considered as a most important factor [4]. Similarly, seed germination is a critical period of the plant growth cycle in hemp, and drought has a negative effect on the growth and yield of hemp in this period [5]. Studies on key cultivation techniques for hemp have mainly focused on variety selection, planting density and plant nutrition [6], and some studies have indicated that salt-alkali or heavy metal stress can influence the germination rate of hempseeds [5], but information regarding drought stress on industrial hemp (or hemp seed germination) is quite limited.

Yunnan Province has the main production area of industrial hemp in China. The land with steep slopes of more than 25 degrees accounts for up to 40% of the total land area of the province. Industrial hemp is mostly planted on hillsides without irrigation facilities. According to the observation by our research team over the years, the planting season of industrial hemp in Yunnan is the driest season during the year. As a result, low and inconsistent emergence, or even no emergence, often occurs because of drought. This kind of situation happens almost every year, and leads to replanting, resulting in waste of labor, material and financial resources, which severely restricts the development of industrial hemp in Yunnan [5]. Therefore, developing cultivation methods to improve hemp drought resistance is in urgent need to develop high-yield and high-quality industrial hemp in Yunnan.

Gibberellins (GAs) are a class of phytohormones and regulate different aspects of plant growth and development through complex biosynthetic pathways [7]. Specific roles of GAs include seed germination, stem elongation, flower initiation, fruit and seed development [8]. Previous studies have shown that exogenous gibberellic acid (GA_3_, one of the most commonly used GAs) promoted seed germination and subsequent seedling growth under various abiotic stress conditions in different plants. GA_3_ has been reported to increase seed germination percentage and seedling growth in *Cicer arietinum* under PEG induced water stress [9,10]. A similar study found that GA_3_ pre-treatments did promote seed germination of *Picea asperata* under drought stress [11]. Seeds pre-treatment with concentrations of 50 ppm GA_3_ at 10 °C for 15 h may be considered as the optimal treatment for *Secale montanum* seeds under drought stress conditions [12]. Shariatmadari et al. showed that under drought stress, treatment with 150 ppm GA_3_ could increase the emergence rate and plant vigor of chickpea seeds in the green house [13]. However, a lack of progress reports on seed pre-treatments effects on seed germination of hemp under drought stress has made it difficult to promote hemp cultivation in arid regions.

Therefore, in present study, seed germination and seedling physiological traits in hemp were examined by applying the treatments of seeds pre-treated with GA_3_ and subjected to drought stress (simulated by PEG-6000). This study also offers a useful tool for the improvement of hempseed germination and has a reference opinion for hemp cultivation in many arid and semi-arid regions of the world.

## 2. Materials and Methods

### 2.1. Plant Materials

Two industrial hemp cultivars, ‘Yunma 1’ (YM) for fiber production and ‘Bamahuoma’ (BM) for seed production, were used in this study. Seeds of YM were bought from Yunnan Industrial Hemp Co., Ltd., Kunming, China and seeds of BM were obtained from Guangxi Academy of Agricultural Sciences, Nanning, China.

### 2.2. Germination Experiment

Hemp seed germination experiment was conducted at 20% (*m/v*) polyethylene glycol (PEG-6000) after soaking seeds in five concentrations of GA_3_ solution (0, 200, 400, 600 and 800 mg/L GA_3_), where 0 mg/L GA_3_ was distilled water. Normal germination (no drought) was used as control and drought stress without seed pre-treating was also designed. Each treatment had 4 replicates arranged in a completely randomized design (CRD). The uniform sized, healthy and intact YM and BM seeds were surface-sterilized with 70% alcohol and washed thoroughly with sterile water several times. The disinfected hempseeds (about 20 g) were soaked in each GA_3_ solution (500 mL) at 20 °C for 8 h and then air-dried for 24 h before being used in the test, all these indices were verified based on a previous experiment [14].

Germination of 120 hempseeds (30 seeds from each replication) representing per treatment were done on two sheets of moist filter paper (previously moistened with either sterile water or 10 mL 20% (*m/v*) PEG-6000 (−0.54 MPa) solution screened in the previous study [15]) in a 12 cm sterile glass Petri dishes. Standard germination test was carried out in an illumination incubator with 65% relative humidity and 20 °C under darkness [16].

### 2.3. Determination of Germination and Morphological Traits

Using the weighing method, sterile water was added to the culture dish every day to keep appropriate PEG concentration in the culture dish. The number of germinated hempseeds was counted and recorded daily for one week. Germination was recorded as the emergence of radical root penetrated the hempseed coat and longer than 1 mm [17]. The germination potential (i.e., the germination rate on the 3rd day after germination) and the germination rate (i.e., the maximum germination rate) of hempseeds were observed and recorded on the 3rd and 7th day after germination, respectively, according to the previous reports [17]. These indexes were calculated according to the following formulas:

Germination potential (%) = (number of germinated hempseeds at 3 days intervals/number of total hempseeds per treatment) × 100

Germination rate (%) = (number of germinated hempseeds at 7 days intervals/number of total hempseeds per treatment) × 100

Seven days after germination, 10 hemp seedlings were randomly selected from each Petri dish to measure radicle length and hypocotyl length of seed.

### 2.4. Determination of Physiological and Biochemical Indices

Seven days after germination, the whole plants of 5 hemp seedlings each from control, 20% PEG, and 20% PEG + GA_3_ were taken to measure the physiological indexes, respectively.

The physiological and biochemical indices, including the activity of antioxidant enzymes (superoxide dismutase, SOD and peroxidase, POD), the content of osmotic regulation substances (soluble protein and soluble sugar) and lipid peroxidation (malondialdehyde, MDA), were determined using the methods described by Wang [18]. SOD activity in hemp seedling was measured by nitroblue tetrazolium (NBT) test. While POD activity was determined by monitoring the increase in absorbance at 470 nm as guaiacol was oxidised. Soluble protein content was measured by Bradford test. The lipid peroxidation level (measured as MDA content) in hemp seedlings was determined by thiobarbituric acid (TBA) test. Soluble sugar content was also determined by TBA test, but the increase in absorbance was measured at 450 nm. All absorbances were taken using a spectrophotometer (UV8000, METASH, Shanghai, China). Both SOD and POD activities were expressed as enzyme U/g (fresh weight, FW). The soluble protein, soluble sugar and MDA concentration (or content) were calculated as mg/g (FW), mmol/g (FW), and umol/g (FW), respectively.

### 2.5. Data Analyses

All results were given as arithmetic means of four replicated measurements with standard deviations except otherwise defined. Data taken for the experiments were processed by two-way analysis of variance (ANOVA) and the means were compared by Duncan’s Multiply Range Test (DM-RT) at 5% significance (*p* < 0.05) in SPSS16.0. Graphical presentation was carried out using SigmaPlot software (for Windows, Version 10.0).

## 3. Results

### 3.1. Effects of GA_3_ Pre-Treatment on Germination Indexes of Hempseeds under Drought Stress

#### 3.1.1. ANOVA Analysis of the Effect of Seed Pre-Treatment by GA_3_ on Germination Indexes of Hempseeds under Drought Stress

A two-way ANOVA showed that hemp cultivars and GA_3_ pre-treatments significantly affected germination rate, germination potential, hypocotyl length, and radicle length of hempseeds (*p* ˂ 0.001) (Table 1). Importantly, their interaction of hemp cultivar and pre-treatment have a significant effect on germination indexes of hempseeds, but their significant levels are different.

#### 3.1.2. Effect of GA_3_ Pre-Treatment on Germination Rate and Germination Potential of Hempseeds under Drought Stress

Drought stress (20% PEG) has significantly affected the germination indexes of YM and BM seeds. Pre-treatment with GA_3_ significantly increased the germination rate and germination potential of YM and BM seeds under 20% PEG treatment (Table 2). Under the condition of pre-treating seeds with different concentrations of GA_3_, the seed germination rate and germination potential of YM and BM increased firstly and then decreased. The highest value of germination rate and germination potential of YM and BM seeds were attained from GA_3_ treatment at 400 mg/L and 600 mg/L, respectively (Table 2).

#### 3.1.3. Effects of GA_3_ Pre-Treatment on Hypocotyl and Radicle Growth of Hempseed under Drought Stress

Drought stress inhibited the seedling hypocotyl and radicle growth from YM and BM seeds. Seeds pre-treatment by GA_3_ alleviated the damaging effect of PEG stress (drought condition) to some extent (Table 2). Compared to drought stress, pre-treating seeds with different concentrations of GA_3_ promoted the hypocotyl and radicle growth of seedlings in both two hemp cultivars. With the increasing concentration of GA_3_, the changing trend in hypocotyl length and radicle length of YM and BM seedlings were the same as that of seed germination rate and germination potential. Maximum hypocotyl and radicle length in YM seedlings were observed at 400 mg/L GA_3_, however, in case of BM maximum hypocotyl and radicle length were gotten at 600 mg/L (Table 2).

### 3.2. Effects of Seeds Pre-Treatment by GA_3_ on Physiology Characteristics in Hemp Seedlings under Drought Stress

#### 3.2.1. Effects of Seeds Pre-Treatment by GA_3_ on Osmotic Regulation Substances in Hemp Seedlings under Drought Stress

**Figure 1:**
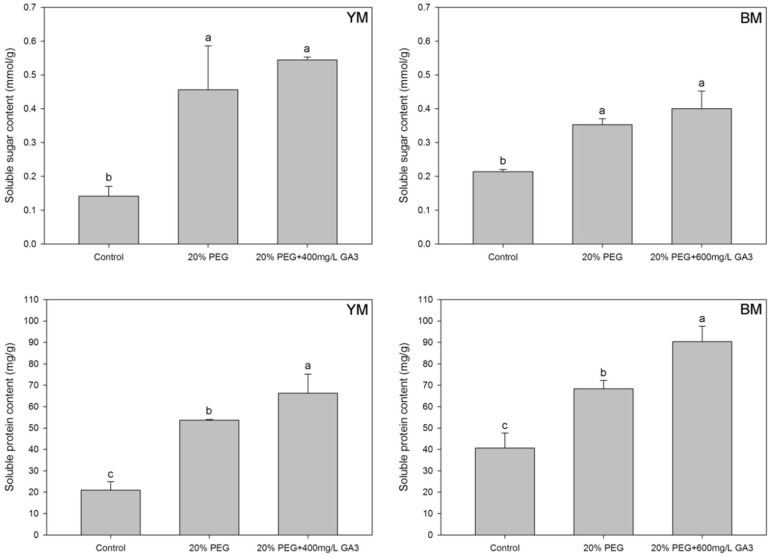
Effects of seed pre-treatment by GA_3_ on soluble sugar and soluble protein contents in hemp seedlings under 20% PEG condition. Different lowercases above the bars show significant difference between treatments (*p* < 0.05). YM: industrial hemp cultivars ‘Yunma 1’; BM: industrial hemp cultivars ‘Bamahuoma’; Control: normal germination with no drought.

Compared to normal germination (control), the level of osmotic regulation substances (soluble sugar and soluble protein contents) in hemp seedlings were increased significantly under 20% PEG induced drought stress (Figure 1). GA_3_ pre-treatments also increased the soluble sugar content in YM and BM seedlings compared to 20% PEG treatment, however, the difference between treatments was not significant. Furthermore, the soluble protein content in YM and BM seedlings were increased significantly at 20% PEG + GA_3_ treatment.

#### 3.2.2. Effects of Seed Pre-Treatment by GA_3_ on Lipid Peroxidation in Hemp Seedlings under Drought Stress

**Figure 2:**
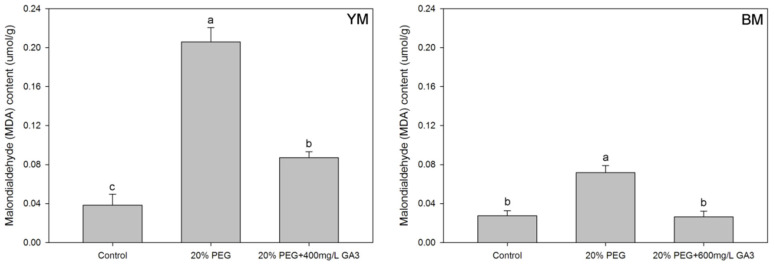
Effects of seed pre-treatment by GA_3_ on the malondialdehyde (MDA) content in hemp seedlings under 20% PEG condition. Different lowercases above the bars show significant difference between treatments (*p* < 0.05). YM: industrial hemp cultivars ‘Yunma 1’; BM: industrial hemp cultivars ‘Bamahuoma’; Control: normal germination with no drought.

Malondialdehyde (MDA) content in hemp (YM and BM) seedlings was significantly increased after PEG treatment (Figure 2). MDA content was increased by 437.6% and 161.1% under 20% PEG in YM and BM seedlings, respectively, compared with normal germination (control). GA_3_ pre-treatment decreased MDA content remarkably in both YM (decreased by 57.6%) and BM (decreased by 63.4%) seedling compared to 20% PEG simulated drought stress condition.

#### 3.2.3. Effects of Seed Pre-Treatment by GA_3_ on Antioxidant Enzymes Activity in Hemp Seedlings under Drought Stress

**Figure 3:**
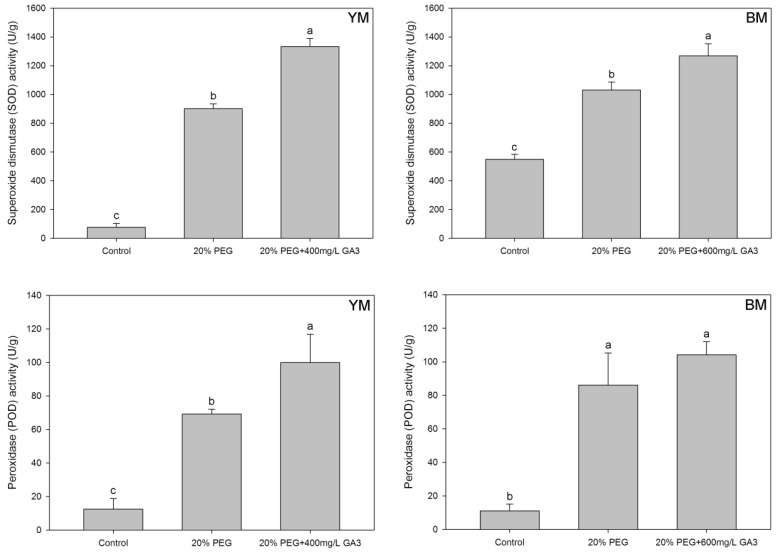
Effects of seed pre-treatment by GA_3_ on superoxide dismutase (SOD) and peroxidase (POD) activities in hemp seedlings under 20% PEG condition. Different lowercases above the bars show significant difference between treatments (*p* < 0.05). YM: industrial hemp cultivars ‘Yunma 1’; BM: industrial hemp cultivars ‘Bamahuoma’; Control: normal germination with no drought.

The activities of antioxidant enzymes (SOD and POD) in hemp seedlings were given in Figure 3. Compared to normal germination (control), SOD activities in hemp seedlings were increased (by 1079.9% in YM and 87.7% in BM) significantly under drought stress induced by PEG treatment. POD activities in hemp seedlings were also increased significantly under drought stress induced by PEG treatment (increased by 453.8% in YM and 680.1% in BM, compared with control).

GA_3_ pre-treatment increased SOD activity in hemp seedlings. SOD activity increased by 48.0% and 23.1% in YM and BM respectively, compared with 20% PEG treatment. GA_3_ pre-treatment also increased POD activity in hemp seedlings. POD activity in GA_3_ pre-treatment seedlings increased by 44.3% and 20.9% in YM and BM respectively, compared with 20% PEG treatment.

## 4. Discussion

Seed germination is sensitive to environmental responses, especially drought stress. Drought not only reduces germination rate of seeds, but also affects the growth of the resulting seedlings in plants [19]. Results of present study showed that PEG-induced drought stress reduced seed germination indexes. Similar results have been reported in tomato [20], maize [21] and rapeseed [22]. However, the seed germination rate and germination potential of both YM and BM drastically decreased and the growth of radicle and hypocotyl in hemp seedling were also delayed under 20% PEG induced drought condition compared to control. These results indicated that hempseed germination is sensitive to drought stress, which has been verified in the field production of hemp in Yunnan or other province in China [5].

As an important endogenous plant growth regulator, GAs plays a vital role in the process of seed germination [23]. Previous reports have suggested that exogenous GAs can reduce the minimum effective exposure time of different germination inhibitors and promote germination rate in flax, sesame, onion and melon seeds [24,25]. In the present study, GA_3_ pre-treated seeds showed higher germination rate and germination potential compared to those that had been not pre-treated under the same PEG condition. Moreover, GA_3_ pre-treatments increased the radicle length and hypocotyl length of hemp seedlings. Thus, seeds pre-treated by GA_3_ did promote seed germination in hemp under drought stress. Generally, the optimal concentration of GA_3_ used for seed soaking is 50~150 ppm in different plants [12,13], but we found that the optimal concentration of GA_3_ for hempseed is 400 mg/L and 600 mg/L in this study. This may be related to the thickness of hemp seed coat. As a matter of fact, exogenous GAs seemed to induce the production and activation of protease and α-amylase in seeds, thus improving the germination ability of seeds [26].

Drought usually leads to osmotic stress, causing an imbalance in the water status of plants [27]. Plants can increase their stress resistance and maintain cell osmotic potential by promoting the effective accumulation of osmotic regulation substances under osmotic stress [27]. Drought stress also triggers the formation of superoxide radical and hydrogen peroxide (H_2_O_2_), which can directly lead to membrane lipids peroxidation and disturbance of plant cell functions. Plants adapt to drought environment by regulating the levels of active oxygen scavenging enzymes such as POD, SOD and catalase (CAT) [28]. Lipid peroxidation (LPO) causes considerable damage to the biological membranes and seems to be the main cause of seed deterioration [29]. As one of the main products of plant lipid peroxidation, MDA accumulation under stress conditions could reflect the degree of damage to plant. A previous study reported that seeds pre-treated with GAs had increased the level of osmotic adjustment, activities of antioxidant enzyme and tolerance to drought stress in *P. asperata* [11]. Soluble sugars and soluble protein were usually utilized early in seed germination as an energy and substance source [11]. In the present study, drought stress increased soluble sugar, soluble protein and MDA contents in hemp seedlings. SOD and POD activities in hemp seedlings were also increased under drought stress. After seeds were pre-treated at different concentrations of GA_3_, the content of soluble sugar and soluble protein increased while MDA content decreased in YM and BM seedling. GA_3_ pre-treated hempseeds showed higher activities of SOD and POD than hempseeds without pretreatment. Findings of the present study showed that seeds pre-treated with appropriate concentration of GA_3_ could further increase the antioxidant enzymes activity and osmotic adjustment ability in hemp seedlings, thus promoting the adaptation of hemp seedlings to drought stress. This also explains why GA_3_ pre-treatments can promote the hypocotyl length and radicle length of hemp seedlings.

Crop cultivars differ in their tolerance (or sensitivity) to stress conditions [30]. Extensive plasticity exists in the levels of drought tolerance across hemp germplasm [16]. In this study, YM and BM are two important industrial hemp varieties released in different provinces of China (Yunnan and Guangxi provinces, respectively). The former is primarily used for fiber production, while the latter is grown for seed. Their seeds can be distinguished from each other by appearance. In particular, YM seeds are larger than BM seeds. Except for the difference of seeds, we also found that the germination rate and germination potential of YM is higher, and the hypocotyl is longer than that of BM under normal germination condition (Table 2). In present study, both YM and BM seeds pre-treated with GA_3_ showed improved tolerance to drought stress, but the optimal pre-treatment concentration of GA_3_ for YM and BM seeds was different, and the concentration of GA_3_ for pre-treatment of YM was lower than that of BM. This was also confirmed in the significant interaction between hemp cultivars and GA_3_ pre-treatments (Table 1). Our previous study revealed that YM was more resistant to drought than BM during seed germination [15]. This was also reflected on the physiological indexes of hemp seedlings grown under PEG-6000 condition, especially the MDA content (reflecting the degree of damage to plant) in BM seedlings was lower than that in YM.

## 5. Conclusions

Results from the final germination indexes (germination rate and germination potential), GA_3_ pre-treatment promoted hempseeds germination. It was possible that the high tolerance of hempseeds to drought stress was associated with its high level of osmotic regulation substances (soluble sugar and soluble protein contents) and antioxidative enzymes (SOD and POD activities), and low MDA content. In this study, we only investigated the effect of GA_3_ on seed germination stage of hemp. The drought resistance effect of exogenous GA_3_ (appropriate concentration) on other growth stages of hemp needs further study. The mechanisms behind, especially the molecular mechanism of GAs promotion of hemp seed germination, remain to be further investigated.

## Figures and Tables

**Table 1:** Two-way ANOVA of the effect of seed pre-treatment by GA_3_ on germination indexes of hempseeds under drought stress.

Effect	Cultivar		Treatment		Cultivar × Treatment	
Parameter	df	F-Value	df	F-Value	df	F-Value
Germination potential	1	75.294 ***	6	37.353 ***	6	4.718 **
Germination rate	1	75.409 ***	6	36.828 ***	6	4.094 **
Hypocotyl length	1	36.973 ***	6	114.749 ***	6	14.576 ***
Radicle length	1	65.094 ***	6	29.768 ***	6	3.317 *

* the significance is denoted as *p* < 0.05; ** *p* < 0.01; *** *p* < 0.001.

**Table 2:** Effect of seed pre-treatment by GA_3_ on germination potential, germination rate, hypocotyl length, and radicle length of hempseeds under drought stress.

Cultivar	Treatment	Germination Potential (%)	Germination Rate (%)	Hypocotyl Length (cm)	Radicle Length (cm)
YM	Control	61.11 + 3.85 a	86.67 + 3.33 a	5.31 + 0.41 a	5.26 + 0.32 a
	20% PEG	16.67 + 3.33 d	35.56 + 1.93 e	1.09 + 0.23 e	3.42 + 0.59 d
	20% PEG + 0 mg/L GA_3_	37.78 + 3.85 c	53.33 + 5.77 d	1.65 + 0.14 d	3.52 + 0.31 d
	20% PEG + 200 mg/L GA_3_	42.22 + 8.39 c	70.00 + 6.67 bc	1.83 + 0.22 d	3.87 + 0.11 cd
	20% PEG + 400 mg/L GA_3_	52.22 + 1.93 ab	78.89 + 5.09 ab	2.89 + 0.12 b	4.79 + 0.04 ab
	20% PEG + 600 mg/L GA_3_	46.67 + 5.77 bc	71.11 + 1.93 bc	2.51 + 0.19 bc	4.35 + 0.41 bc
	20% PEG + 800 mg/L GA_3_	43.33 + 6.67 bc	68.89 + 6.94 c	2.41 + 0.34 c	3.94 + 0.17 cd
BM	Control	51.11 + 5.09 a	80.00 + 6.67 a	3.33 + 0.41 a	5.42 + 0.89 a
	20% PEG	18.89 + 3.85 e	31.11 + 6.94 d	1.35 + 0.06 d	1.99 + 0.37 d
	20% PEG + 0 mg/L GA_3_	21.11 + 3.85 de	40.00 + 11.55 cd	1.44 + 0.22 d	2.18 + 0.61 d
	20% PEG + 200 mg/L GA_3_	24.44 + 1.93 cde	44.44 + 7.70 cd	1.58 + 0.11 d	2.70 + 0.26 cd
	20% PEG + 400 mg/L GA_3_	27.78 + 5.09 cd	46.67 + 5.77 bc	2.28 + 0.23 bc	3.25 + 0.24 bc
	20% PEG + 600 mg/L GA_3_	37.78 + 5.09 b	58.89 + 3.85 b	2.54 + 0.14 b	3.78 + 0.24 b
	20% PEG + 800 mg/L GA_3_	30.00 + 3.33 c	45.56 + 6.94 bc	2.11 + 0.10 c	2.74 + 0.29 cd

Different lowercase letters in the same column in each cultivar indicate a significant difference between treatments (*p* < 0.05).

## Data Availability

Data are contained within the article.
